# Mobile Device–Based Electronic Data Capture System Used in a Clinical Randomized Controlled Trial: Advantages and Challenges

**DOI:** 10.2196/jmir.6978

**Published:** 2017-03-08

**Authors:** Jing Zhang, Lei Sun, Yu Liu, Hongyi Wang, Ningling Sun, Puhong Zhang

**Affiliations:** ^1^ The George Institute for Global Health at Peking University Health Science Center Beijing China; ^2^ Beihang University Beijing China; ^3^ The People’s Hospital, Peking University Beijing China

**Keywords:** mEDC, electronic data capture, mobile data capture, mhealth, randomized controlled trial, clinical research

## Abstract

**Background:**

Electronic data capture (EDC) systems have been widely used in clinical research, but mobile device–based electronic data capture (mEDC) system has not been well evaluated.

**Objective:**

The aim of our study was to evaluate the feasibility, advantages, and challenges of mEDC in data collection, project management, and telemonitoring in a randomized controlled trial (RCT).

**Methods:**

We developed an mEDC to support an RCT called “Telmisartan and Hydrochlorothiazide Antihypertensive Treatment (THAT)” study, which was a multicenter, double-blinded, RCT, with the purpose of comparing the efficacy of telmisartan and hydrochlorothiazide (HCTZ) monotherapy in high-sodium-intake patients with mild to moderate hypertension during a 60 days follow-up. Semistructured interviews were conducted during and after the trial to evaluate the feasibility, advantage, and challenge of mEDC. Nvivo version 9.0 (QSR International) was used to analyze records of interviews, and a thematic framework method was used to obtain outcomes.

**Results:**

The mEDC was successfully used to support the data collection and project management in all the 14 study hospitals. A total of 1333 patients were recruited with support of mEDC, of whom 1037 successfully completed all 4 visits. Across all visits, the average time needed for 141 questions per patient was 53 min, which were acceptable to both doctors and patients. All the interviewees, including 24 doctors, 53 patients, 1 clinical research associate (CRA), 1 project manager (PM), and 1 data manager (DM), expressed their satisfaction to nearly all the functions of the innovative mEDC in randomization, data collection, project management, quality control, and remote monitoring in real time. The average satisfaction score was 9.2 (scale, 0-10). The biggest challenge came from the stability of the mobile or Wi-Fi signal although it was not a problem in THAT study.

**Conclusions:**

The innovative mEDC has many merits and is well acceptable in supporting data collection and project management in a timely manner in clinical trial.

## Introduction

An adequate central data management is crucial and indispensable in order to manage data capture, data integration, data storage, and data transfer in clinical trials [1]. However, paper-based data collection has considerable challenges of data management and requires additional time for double data entry, cleaning, and analysis [2]. In several cases, a conventional central data management comprising Web applications for data capture and central databases provides a suitable solution, but these are often limited by lack of reach and widespread applicability [3]. For example, since such tools are made available in an internal secured network, data capture can only be conducted from personal computers within this network. Moreover, data collection from subjects often takes place in rooms where a designated personal computer is not accessible. More importantly, most studies relying on computer-based data capture tools are not required to submit captured data in real time during collection.

In the past decade, certain mobile device–based tools such as personal digital assistants (PDAs) [4,5] and mobile phones [6,7] have been used to collect and manage data. Such tools show great performance and qualities including high-quality data, more effective training to the staff, user friendliness, and cost effectiveness. More importantly, in recent decades, access to the Internet using wireless and mobile communication technologies has tremendously increased and has been adapted for use in field research settings, particularly in medical research, because of the ease of transferring data in real time and convenience owing to the portability of mobile devices. Low- and middle-income countries such as China and India lack the infrastructure to accommodate adequate fixed-line Internet access in rural areas, and cellular networks allow access to telecommunications in such regions with limited Internet access [8]. China has the largest and fastest growing mobile Internet population, with 668 million people using the Internet (89% via mobile phones) as of June 2015 [8], which supports effective implementation of studies using mobile devices in China.

Nevertheless, mobile technology has been barely used to support implementation of pharmaceutical randomized controlled trials (RCTs). Compared with traditional computer-based clinical trial management systems, mobile device–based methods for clinical trial reporting have distinct features that can ensure data transfer and exchange, telemonitoring, and project management in real time.

From 2014 to 2015, we conducted a multicenter, randomized, double-blinded, parallel-controlled trial with the aim of comparing the efficacy of monotherapy with two types of medications in high-sodium-intake patients with mild to moderate hypertension (*T* elmisartan 40 mg/day and *H* ydrochlorothiazide [HCTZ] 25 mg/day *A* ntihypertensive *T* reatment study or the THAT study). During implementation of the THAT study, we developed an innovative, mobile device–based electronic data capture (mEDC) system to support data capture, monitoring, and project management. In addition, we performed a designated qualitative evaluation based on experiences of various types of users to determine whether mEDC can effectively facilitate data collection, project implementation and management, and real-time telemonitoring.

## Methods

### Design and Features of mEDC

Eligible participants for the THAT study included adults aged ≥18 years with mild to moderate hypertension not using antihypertensive or blood pressure (BP)–altering medications for at least one week. Overall, 1333 participants were recruited at clinics from 14 rural county hospitals, randomly divided into the telmisartan and HCTZ treatment groups, and followed up on the 15th, 30th, and 60th day after enrollment. Dummy telmisartan and dummy HCTZ were used to ensure double blinding. The primary outcomes included BP decrease, BP control rates, fasting blood glucose (FBG), hypokalemia, and adverse events (AEs).

The mEDC system was developed to help users (doctors, clinical research associates [CRAs], project managers [PMs], and data managers [DMs]) in data collection, telemonitoring, and project management. The mEDC, consisting of two primary components (an app installed in mobile phones and a server-based clinical trial database) was designed in accordance with the guidelines laid down by the International Conference on Harmonization Technical Requirements for Registration of Pharmaceuticals for Human Use Good Clinical Practice (ICH-GCP) [9]. We prospectively wrote design specifications to describe structure of the mEDC, and a vendor independently developed this app following our specifications. We also developed a test plan based on these specifications to justify validation of the mEDC system. In order to ensure regulatory compliance, we referred to three additional guidelines focusing on EDC systems for RCTs while designing mEDC: Good Clinical Data Management Practices proposed by the Society for Clinical Data Management (SCDM) [10], Guidance for Industry Computerized Systems Used in Clinical Investigations represented by the Food and Drug Administration [11], and Guidance for Industry Part 11 of Title 21 of the Code of Federal Regulations on Electronic Records and Electronic Signatures (21 CFR 11) [12]. Before implementing the primary study, we invited two independent research staff to validate the mEDC system. They entered a batch of data (approximately 50 simulated patients) into the system to test not only the clinical database validation, including data entry screen testing, data checking routines (eg, range and format), testing of data verification functions, and data transferring (remote data entry) but also trial-specific validation of variables such as name, label, type, and randomization of subjects [10]. Table 1 summarizes the major supportive functions and features of mEDC.

**Table 1:** Functions and features of mobile device–based electronic data capture (mEDC) and relevant requirements as per electronic data capture (EDC) guidelines.

No.	Functions and features of mEDC^a,b^		Requirements per EDC^a,c^ guidelines and comments
1	**Site equipment and administration**		
		Logistic management	Should be maintained either onsite or be remotely accessible through electronic files (IV-B: SOP^d^ [11])
		Role allocation	Access must be limited to authorized individuals (IV-D-1: Limited access [11])
2	**Patient recruitment and data collection**		
		Screening	These 6 parts follow “Guidance for Industry Computerized Systems Used in Clinical Investigations” [11]: each step will be used to create, modify, maintain, archive, retrieve, or transmit source data (IV-A: Study Protocol [11]), ensure the system’s date and time stamps are accurate (IV-D-3: Date/Time stamps [11]), alert the user if data are out of acceptable range, and should not automatically enter data (IV-F-1: Direct Entry Data [11])
		Data collection at baseline	
		Centralized randomization	
		Data collection during follow-up	
		BP^e^ measurement	
		Record of adverse events	
3	**Patient referral and reference data**		
		Biological sample collection	The same as No. 2 (same as above)
		Medicine prescription	
4	**Remote validation of data**		To assure that data are reliable, complete, and accurate (Data entry and data processing [10]); keeping track of all changes made to data in electronic records (IV-D-2: Audit trails [11]); audit trails can be particularly appropriate when users are expected to create, modify, or delete regulated records during normal operation (III-C-2: Audit trails [12])
5	**Data storage and management**		A copy of the data should be maintained at another location, typically at the clinical site (IV-C: Source documentation and records retention [11], data entry and data processing [10], measuring data quality [12]); should be accessible only by using their own password (IV-D-1: Limited access [11]) Procedures and controls should be put in place to prevent altering, browsing, querying, or reporting of data via external software applications (IV-E: External security safeguard [11]) Provided to FDA^f^ and should fully describe and explain how source data were obtained and managed and how electronic records were used to capture data; have dependable system documentation (IV-F-2: Retrieving data [11]) Create and preserve electronic records, sufficient backup, and recovery procedures (IV-F-4: System controls [11])
6	**Institutional Review Board operations**		
		Clicking a photograph of informed consent forms to ensure completion of IRB^g^ operations	No requirements for IRB operations in the guidelines
7	**Other functions and features**		
		Quality control for BP measurements	No requirements for quality control of BP measurements in the guidelines
		Quality control for consistence of medicine codes	Ensuring processes are defined to integrate laboratory and other non-CFR^h^ data with the data from the eCRF^i^ (Electronic Data Capture Principles [10])
		Project progress and status reports	Automate generation of reports on metrics and project status to facilitate project or site or patient management (Electronic Data Capture Principles [10])

^a^Mobile phone Redmi Note (5.5 inch, 1GB RAM, 32GB ROM, 4G Dual SIM, Android system v4.2) was used as the mobile device. Moreover, mEDC can be installed and used in mobile phones from most other brands that are based on the Android system.

^b^mEDC: mobile device-based electronic data capture.

^c^EDC: electronic data capture.

^d^SOP: standard operating procedure.

^e^BP: blood pressure.

^f^FDA: Food and Drug Administration.

^g^IRB: Institutional Review Board.

^h^CFR: Code of Federal Regulation.

^i^eCRF: electronic case report form.

#### Site Equipment, Training, and Administration

At each study site, doctors were required to take some photographs using the camera of their mobile phones to verify that they had completed the role allocation and internal training for study preparation. Delivery of all site-specific materials such as study medicines, tubes for biological samples, and other materials, both to and from each site, was confirmed by the doctors, and the materials were photographed for record. The PMs remotely checked these photographs, which were required to be uploaded in the mEDC app, and sites would initiate the process after receiving confirmation from PMs. The study protocol and standard operating procedures (SOPs) were stored in the cellphones, allowing convenient access of these instructions.

#### Patient Recruitment and Visits

Once patients signed informed consent forms, doctors collected necessary information using mEDC, following which the server immediately returned the results to verify whether or not the patients met the inclusion criteria. After baseline information had been collected, unique and structured randomization codes were allocated to all patients. Simultaneously, mEDC allocated study medicines and test tubes bearing the same codes as assigned to the patients. Each patient was assigned a specific time slot for site visits. mEDC helped doctors to arrange visits through 2 automatic pop-up options: sending a standard short message and calling the patient immediately. Patients who had opted to receive a short message received a message with the name of the doctor indicating when he or she should visit the doctor, if breakfast and medicines should be taken, and if the leftover medicine should be brought along. Patients who had difficulty in receiving or reading messages were contacted over telephone on their primary or alternate contact numbers that had been collected at the first visit. During visits, all data transactions between mEDC at the site and the central study server were automatically stamped with identification regarding who provided the information, when it was provided, from where, and how.

#### Centralized Randomization

THAT study was a double-blinded study with stratified block randomization. Each box of study medicines that contained a genuine medicine and the other dummy medicine was allocated a specific code according to the randomization list in advance. The randomization list was generated previously with gender and systolic blood pressure (SBP ≥160 mmHg) as stratified variables and a random block size of 4. A range of medicine boxes with sequential codes of randomization were delivered to each site. Site names and the matched codes list were uploaded to the mEDC server so that doctors could identify the specific medicines according to the code returned by mEDC during randomization. Biological sample tubes and other study materials with the same codes were delivered to the same site. This guaranteed that a patient had received study medicines and materials with their unique code. Doctors could trigger an unblinding procedure for patients in emergency via mEDC during follow-ups.

#### Quality Control for Key Procedures

BP measurement was the primary outcome of the THAT study. Doctors received strict training and were provided with uniform digital sphygmomanometers. In practice, the entire procedure was automatically controlled by mEDC. Before measurements, mEDC required doctors to click photographs to record the posture of patients at the time of measurement. During measurements, it instructed doctors to measure BP for 3 times at 2-min intervals.

Another key point for quality control was the consistence of medicine codes, that is, the codes that mEDC allocated to patients had to be the same as the codes that were on the medication boxes. Doctors were required to click photographs to record the codes on the medication boxes, and CRAs checked to ensure this consistency.

#### Patient Referral

At the end of the visit, doctors instructed patients to collect a blood or urine sample, and a message would pop-up on the mobile phone of the nurses responsible for blood or urine sample collection as follows: “Patient xxx with code yyy will arrive for sample collection. Please make sure to use the tubes with the same code.” A similar message would be sent to the study medication distributors to support study medication distribution. The entire process was designed to ensure consistency of patients’ codes across all procedures.

#### Remote Validation of Data

Similar to traditional computer-based EDC systems, most variables in the THAT study were checked for missing values, range of variable values, outliers, and irrationalities during data entry. However, in contrast to the traditional EDC systems, data entry and validation in the mEDC was performed at the time point of patients’ site visit rather than at a later time point. In addition, by relying on camera technique, mEDC could ask doctors to provide evidence by clicking photographs for key information. Photographic evidence was collected for consistency of patient identification on different materials at all 4 visits, BP measurements, and other procedures. For example, doctors were required to not only enter BP measurements but also to click a photograph for interfaces of digital sphygmomanometers and upload them on the server. The consistency between these recordings would be remotely checked by a CRA. In addition, CRAs remotely supervised the recruiting progress, data validity, missing data, and risk of loss to follow-up through mEDC and in a timely manner. Logistical checking could also be done based on the database temporarily downloaded from the server. We did not develop Web-end to track modification for each variable but used a specially designed database to record any modifications. The mEDC DM provided frequent reports on modifications by running a program based on the database.

#### Data Storage and Management

Owing to the need for tracking patients during referral and sending follow-up reminders, certain private data such as names and telephone numbers were recorded in mEDC. During data or project management, personal information of participants was shielded from DMs and CRAs, and the deidentified data could only be transferred to researchers and statisticians for data analysis. No hardcopy CRFs were used in the THAT study. Once submitted, collected data was then transferred to the server (located in Beijing) without any information stored in mobile phones. Instant photocopies of key information or records such as BP readings, codes of study medications, and biomedical samples were also uploaded to the server. All the data stored in the server was quarterly backed up to a designated local laptop. Secure Hash Algorithm 512 (SHA-512) [13], a more efficient and secure algorithm, was implemented to ensure that all data were stored in the server and not in local mobile phones. Only authorized doctors could log in to mEDC and access the information collected by them. During the process of data transfer, cryptographic net key was initiated when an investigator logged in. To ensure safe data exchange, the “HTTPS” was utilized in the linkage between the terminal device and the server, which formed a safe circle to prevent stealing or misuse of data.

#### Institutional Review Board Operations

As part of the study SOP, doctors were required to click a photograph of the page with signatures and submit it to the server, which could help CRAs verify patients’ recruitment because signatures of patients and doctors were essential as per the recommendations of 21 CFR 11 [12].

#### Project Progress and Status Reports

Doctors could use the recruitment number of patients to confirm whether they had completed their follow-up or not, and mEDC would send reminders to doctors regarding the same. On the other hand, CRAs and PMs could easily track the progress at each site along with statistical summaries of each site, and they would receive notifications when any AE occurred.

### Operation of mEDC

**Figure 1:**
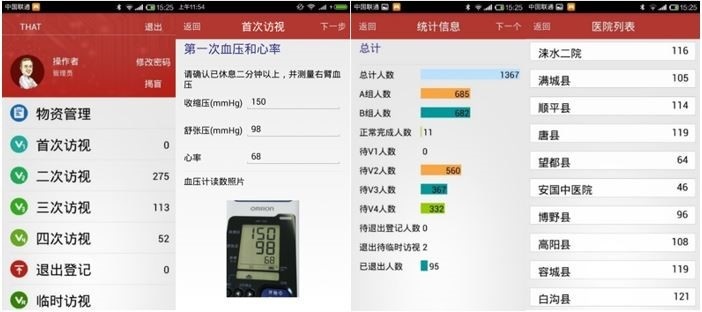
Certain interfaces of mobile device–based electronic data capture (mEDC).Fig 1-1 Root directory for investigators and CRA to manage materials, visits, and quit register; Fig 1-2 Interface for investigators to input blood pressure and upload photo evidence of electronic sphygmomanometer screens with 2 minutes interval for each of three sequential blood pressure measurements;Fig 1-3 Current reports of recruitment and follow-ups for site investigators;Fig 1-4 Current reports of all sites for CRA and principle investigators.

Certain interfaces are presented in Figure 1.

**Figure 2:**
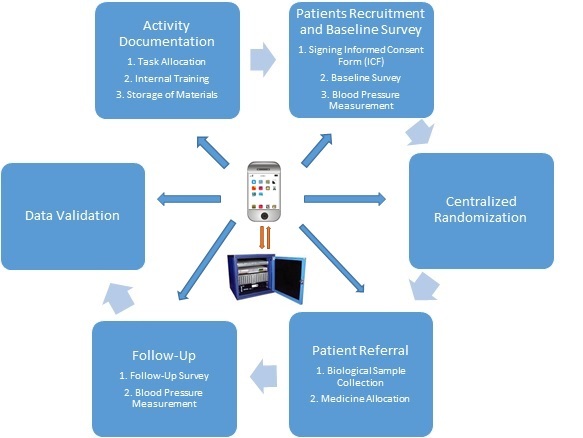
Functions and operations of mobile device–based electronic data capture (mEDC).

Every step of study implementation and the actions mEDC supported are presented in Figure 2.

### Qualitative Evaluation

An intensified sampling method was used to select participants in different roles involved in the THAT study to evaluate the feasibility of mEDC, including 1-2 doctors at each hospital, 1 CRA, 1 PM, and 1 DM.

Semistructured qualitative interviews were conducted by 2 trained interviewers during and after the clinical trial. The structured questions included (1) individual general information and infrastructure conditions, (2) time taken for data collection at each visit, and (3) user satisfaction scores. Satisfaction scores ranged on a scale of 0-10, with a score of 10 indicating “most satisfied,” and a score of 0 indicating “most dissatisfied.” In-depth personal interviews were conducted to explore individuals’ understanding and feelings of mEDC, which covered (1) evaluation of practical use of mEDC, (2) willingness of using such an app in the future, and (3) experience of security in data storage and transfer. Simultaneously, interviewers objectively assessed how users maneuvered the app during project implementation. Following description of interview guidelines, all participants were interviewed face-to-face, and their responses were recorded. In addition, 53 patients were interviewed through telephone calls to enquire about their experience when visited by doctors through mEDC.

Nvivo 9.0 was used for data extraction and classification after transcribing and restructuring all the records into *.doc* format. A thematic framework method was used to analyze the interview transcripts [14]. Two independent groups separately conducted data extraction and classification, and any discrepancies between the two groups were resolved by consensus. Because a non-English interview guide was used, back-translation [15] was used to ensure that the original information provided by the interviewees was accurate and reliable.

### Ethical Approval

This study received approval from the Peking University at Medical Health Science Center Ethics Committee (ref: IRB00001052-14039). Signed informed consent was obtained from all participants before the qualitative interviews.

## Results

### Telecommunication Signal Coverage and Characteristics of Participants

Table 2 summarizes the demographic and other characteristics of the participants. All the study hospitals had access to 3G or 4G and/or WiFi signal.

**Table 2:** Demographic and other characteristics of all participants.

Characteristics	Doctors (n=24)	Patients (n=53)	CRA^a^ (n=1)	PM^b^ (n=1)	DM^c^ (n=1)
Age (years), mean (SD^d^)	40.6 (4.2)	53.5 (5.2)	26	34	37
Male (n)	10	23	0	0	1
Years of work experience, mean (SD)	11.2 (5.1)	-	2	10	10
Prior clinical trial experience, n (%)	7 (29.2)	-	1 (100.0)		1 (100.0)
Possession rate of any cellphone, n (%)	24 (100.0)	40 (76.0)	1 (100.0)		1 (100.0)
Possession rate of mobile phone, n (%)	24 (100.0)	13 (24.0)	1 (100.0)		1 (100.0)

^a^CRA: clinical research associate.

^b^PM: project manager.

^c^DM: data manager.

^d^SD: standard deviation.

### Trial Implementation, BP Measurements, and Time Consumption for Data Collection

Overall, 1333 out of a total of 2130 participants were recruited in 14 Hebei county hospitals from October to December 2014; 670 participants were randomly allocated to the telmisartan group and 663 to the HCTZ group. At the end of the study, 1037 participants had completed three follow-up visits in 2 months. The average SBP/DBP (diastolic blood pressure) reduction in telmisartan group and HCTZ group was 12.8/7.2 and 11.5/5.3 mmHg, respectively. As an example, the details of 3 BP measurements at baseline are described in Table 3.

**Table 3:** Three BP measurements at baseline (n=1188).

BP	First measurement, mean (SD)	Second measurement, mean (SD)	Third measurement, mean (SD)	The average of later two measurements, mean (SD)
SBP^a^	160.1 (10.6)	155.3 (10.1)	153.1 (11.6)	154.1 (11.1)
DBP^b^	99.3 (8.9)	94.5 (8.7)	91.3 (9.3)	92.8 (8.8)

^a^SBP: systolic blood pressure.

^b^DBP: diastolic blood pressure.

The entire questionnaire had 141 questions including 54 questions for baseline visit and 29 questions for each of visits 2-4, excluding the 15 questions in the severe AEs form. As estimated by the 24 doctors, the average time used for data collection directly through mEDC was 53.0 (SD 5.3) min for all the 4 visits. All doctors considered the total time taken was reasonable for them, and most patients could bear the standby period. No patient had any complaints related to the procedure when interviewed through mEDC.

I think I can afford the average time spent on visiting one patient. The study did not affect my routine work. Although patients usually attend visits here in the morning, I spent only around 10 minutes per patient to complete each visit.

Doctor, female, 46 years

*I feel I experienced a satisfactory process. Following reminders, my doctor told me how to conduct every step during the visit. The time spent on completing each visit was considerably short.* [Patient, female, 51 years]

### Experience of Doctors

All doctors mentioned that mEDC was very convenient and could help them complete the visits smoothly. They were willing to use mEDC to implement the THAT study, although the current mEDC app still needs to be upgraded, such as addition of a function of patient indexing. Moreover, they expressed that they would be interested in participating in other clinical trials with mobile phones in the future. Table 4 summarizes the satisfaction scores.

**Table 4:** Satisfaction scores with mobile device–based electronic data capture (mEDC) from 24 doctors.

No.	Procedures		No. of participants (nonresponse^a^/total^b^)	Satisfaction score (median, range)
1	**Procedure of implementation**			
		Inclusion and exclusion	0/18	9 (8-10)
		Informed consent	0/18	9 (8-10)
		Data collection at each visit	0/18	9 (8-10)
		Randomization	0/18	9 (8-10)
		Collection of biological samples	0/18	9 (7-10)
		Medicine delivery	0/18	9 (7-10)
		Appointment for next visit	1/18	8 (6-9)
2	**Project management**			
		Accounts management	0/18	9 (8-10)
		Logistics	1/18	9 (8-10)
		Patient indexing	0/18	8 (6-9)
3	**Quality control**			
		Reminder at each step	0/18	9 (8-10)
		Key point control	0/18	9 (8-10)
4	**Others**			
		Training for using mEDC^c^	2/18	9 (8-10)
		Wireless Internet	0/18	8 (6-9)
	Overall average score			9 (6-10)

^a^Nonresponse: number of interviewees who had no response.

^b^Total: total number of participants who attended the qualitative evaluation.

^c^mEDC: mobile device-based electronic data capture.

Although the study involved several steps, such as visit, blood pressure measurement, biological sample collection, medicine description, and visit appointment, the smartphone assisted me to complete every task without any trouble, and I only needed to follow the tips provided by mEDC. I can maneuver the system easily, even though I am not familiar with the functions of my smartphone.

Doctor, male, 44 years

Although I do not have prior experience in a clinical trial, I think that mEDC helped me collect the data, manage my patients, remind me of what the next step was, and when I should follow up my patients. It made me efficient and confident to execute my job. If possible, I am keen to be part of other studies with smartphones in the future. I hope that for those patients who completed their visits, an indexing function should be available in mEDC, which can help them find their doctor (me) easily.

Doctor, male, 40 years

### Experience of CRAs and PMs

CRAs and PMs expressed that this was their first experience of using mEDC to monitor or manage a pharmaceutical clinical trial. They all believed that mEDC offered some creative functions that were not available in traditional computer-based clinical trials, such as material dispatching and tracking, remote monitoring and validation in real time, and supervising the progress of the trial. mEDC is a promising tool for use in future pharmaceutical clinical trials.

I could monitor quality remotely through mEDC, viewing the data instantly when doctors uploaded their data. As soon as I found that any figure or photograph was possibly wrong, like blood pressure measurements, I would contact them as soon as possible. I also noticed that compared with traditional clinical trials, doctors did not easily procrastinate, since mEDC required the doctors to correct mistakes in a timely fashion, and this feature can help reduce my workload.

CRA, female, 26 years

I believe that it is a very good tool for pharmaceutical clinical trials. The data uploading in real time, the smooth operation, and the user-friendly interface are highly impressive features. Although this was my first experience using such a cellphone-based system, I believe that it can be promoted in the field of randomized controlled trials in the future. Compared with traditional trials, especially for those trials in which the data source is not from health records, like the THAT study, this mEDC could help us manage the project and control quality in real time. The most impressive detail is that the function of the 2-minute interval between two blood pressure measurements can systematically ensure that doctors follow the SOP, so as to reduce potential bias.

Project manager, female, 34 years

### Experience of DMs

DMs also believed that mEDC was safe and using it could help reduce workload during the process of data cleaning.

With almost 10 years of experience focusing on clinical data management, I consider that the whole structure of mEDC is very good and reasonable, especially the safety. For example, “HTTPS” can make sure that the process of data transfer is safe. Moreover, the server is cryptographic, and only people who know the password can have access to it. Spontaneously, real-time uploading enables data not only to be stored in phones but also to be stored on the server, which can avoid information loss to an extremely high extent. More importantly, error correction of mEDC in real time considerably reduces workload at the time of data cleaning. However, a limitation is that there is no modification track in mEDC interfaces, which would help doctors recall work experience. I hope to see this function in the next version of mEDC.

Data manager, male, 37 years

## Discussion

### Principal Findings

Mobile device-based technology or app is generally used in several domains [3], including for data collection and reporting [6]; however, at present, project management in pharmaceutical clinical trials scarcely involves mobile devices. The THAT study was successfully completed under management of mEDC, suggesting that data collection and management using mEDC was technologically feasible using a mobile phone in this study. The positive feedback of the users confirmed the feasibility of mEDC, which also establishes substantial confidence about the utilization of mEDC in future pharmaceutical clinical trials.

The significant and specific features of mEDC are data collection, monitoring, and project management in real time. Although computer-based trials proposed that they could recruit patients and collect data in real time by using Internet [16], in most cases, doctors were more likely to rely on transferring data by means of paper-to-computer when they captured clinical data, particularly in trials wherein data sources involved health records. mEDC made the aforementioned process more straightforward owing to portability of mobile phones and was more beneficial in entering data directly in real time. Under such situations, PMs can remotely monitor the progress of the project in real time. Theoretically, mEDC can reduce the time required for data capture because doctors use mobile phones to collect information directly instead of recording such information in paper-based CRFs in the first place. Although we did not compare the effectiveness between these two approaches, evidence available from several previous studies suggests that using mobile phones for data collection, instead of with pen-and-paper, eliminated data recording and entry errors, had similar interrater reliability, and took an equal amount of time per interview but with no second entry [17].

The design for quality control and data validation through mEDC was unprecedented in this time of computer-based clinical trials, particularly for validation of the primary outcome (BP measurement), which could assure that the BP of every patient was accurately measured. The photographic evidence of patients’ seated posture and reminders for 2-min intervals between every two measurements could control doctors to follow the SOP of BP measurement, whereas the screenshots of the digital sphygmomanometer after each BP measurement could help CRAs check the consistency between BP values and screenshots, which can be difficult to achieve in computer-based trials. This type of design can help CRAs remotely conduct site monitoring, reduce extremely inaccurate information, improve data quality, and reduce the workload of DMs at the time of data cleaning. At the same time, Table 3 shows that among three average BP measurements recorded at baseline, the first BP measurement was higher than the other two, the second one was moderate, whereas the third one was the lowest; however, the latter two measurements exhibit a stable trend, which is in concordance with findings from a previous study [18]. This, in a way, validates the reliability of data transferred using mEDC.

From the perspective of doctors, mEDC could be used correctly and smoothly in county hospitals because the user-friendly operation system and succinct screen could help them complete a high-quality clinical trial through a built-in automated procedure of the mobile phone, regardless of prior clinical trial experience. Time consumption analysis and qualitative interviews also indicated that mEDC was easy to operate, and it did not impose any additional workload on doctors. This portable data capture tool not only benefitted doctors but also benefitted CRAs with convenience of data validation.

With regard to security, like SHA-512, which guarantees safety of data transfer [13], HTTPS assured secure connection of the mobile phone with the server and prevented it from being attacked by hackers. The central server could encrypt all transmissions and restrict each individual to appropriate access to data and operations on data.

### Limitations

This study had certain limitations. The biggest concern was the signal shielding and stability of the cellular network. Although mobile network is more popular than fix-line Internet in China [3], the signal could probably be impacted due to shielding by the environment, such as in indoor areas of certain buildings with reinforcement concrete frames. The THAT study was a phase IV clinical trial, which is relatively simpler than phase I-III clinical trials; thus, the mEDC algorithm would probably need to be designed in a more complex fashion if it is to be utilized in other phases of a clinical trial. Moreover, whether available mobile phones can process algorithms that are more complicated remains unknown. The current version of mEDC was not perfect because of limited design time. For example, queries had not been designed in mEDC; therefore, any track changes were not shown on the screen of the mobile phone. Although the track changes could be recorded in the server, doctors could not identify which data were modified; this resulted in some inconveniences. BP measurements were to be manually filled in mEDC but could not be automatically transferred via Bluetooth. Furthermore, doctors were not authorized to record electronic signatures during IRB operations but rather had to record photographs of the informed consent form pages with signatures for data retention. Nonetheless, this limitation will be addressed and fixed in an upcoming updated version of this app.

### Conclusions

The mobile device-based data capture and project management system, mEDC, could help doctors complete a phase IV pharmaceutical clinical trial and was feasible for management of this trial. Moreover, doctors expressed their willingness to use this tool for study implementation. The validity, reliability, real-time feature, and user friendliness of mEDC are beneficial not only for doctors without clinical trial experience but also for CRAs and PMs. Taken together, there is a possibility for mEDC to be used in other pharmaceutical clinical trials in the future.

## Abbreviations

AEs: adverse events
BP: blood pressure
21 CFR 11: Guidance for Industry Part 11 of Title 21 of the Code of Federal Regulations on Electronic Records and Electronic Signatures
CRAs: clinical research associates
DMs: data managers
eCRF: electronic case report form
EDC: electronic data capture
FBG: fasting blood glucose
FDA: Food and Drug Administration
HCTZ: hydrochlorothiazide
ICH-GCP: International Conference on Harmonization Technical Requirements for Registration of Pharmaceuticals for Human Use Good Clinical Practice
IRB: Institutional Review Board
mEDC: mobile device–based electronic data capture
PMs: project managers
SBP: systolic blood pressure
SCDM: Society for Clinical Data Management
SD: standard deviation
SHA-512: Secure Hash Algorithm 512
SOPs: standard operating procedures
